# DNA damage in the kidney tissue cells of the fish *Rhamdia quelen* after trophic contamination with aluminum sulfate

**DOI:** 10.1590/S1415-475738420140327

**Published:** 2015

**Authors:** Tatiane Klingelfus, Paula Moiana da Costa, Marcos Scherer, Marta Margarete Cestari

**Affiliations:** Departamento de Genética, Universidade Federal do Paraná, Curitiba, PR, Brazil

**Keywords:** fish, metal contamination, chromosomal abnormalities, comet assay

## Abstract

Even though aluminum is the third most common element present in the earth's crust, information regarding its toxicity remains scarce. It is known that in certain cases, aluminum is neurotoxic, but its effect in other tissues is unknown. The aim of this work was to analyze the genotoxic potential of aluminum sulfate in kidney tissue of the fish *Rhamdia quelen* after trophic contamination for 60 days. Sixty four fish were subdivided into the following groups: negative control, 5 mg, 50 mg and 500 mg of aluminum sulfate per kg of fish. Samples of the posterior kidney were taken and prepared to obtain mitotic metaphase, as well as the comet assay. The three types of chromosomal abnormalities (CA) found were categorized as chromatid breaks, decondensation of telomeric region, and early separation of sister chromatids. The tests for CA showed that the 5 mg/kg and 50 mg/kg doses of aluminum sulfate had genotoxic potential. Under these treatments, early separation of the sister chromatids was observed more frequently and decondensation of the telomeric region tended to increase in frequency. We suggest that structural changes in the proteins involved in DNA compaction may have led to the decondensation of the telomeric region, making the DNA susceptible to breaks. Moreover, early separation of the sister chromatids may have occurred due to changes in the mobility of chromosomes or proteins that keep the sister chromatids together. The comet assay confirmed the genotoxicity of aluminum sulfate in the kidney tissue of *Rhamdia quelen* at the three doses of exposure.

## Introduction

Aluminum is the third most common element in the earth's crust (Nayak, 2002) and can be found in small quantities in several types of food (Koivistoinen, 1980). Small amounts of aluminum are released from cooking utensils and are dissolved in the food, particularly when the food is acidic. Furthermore, aluminum compounds are often used in water purification (Lione, 1983), as catalysts in the chemical and paper industries, in the dyeing of textiles, and in other applications (Ganrot, 1986). Despite the extensive use of aluminum, the information related to its toxicity is scarce (Lankoff *et al.*, 2006).

The toxicity of metals, including aluminum, is an extremely complex matter (Guthrie and Perry, 1980; Hamond and Beliles, 1980) that is related to at least three types of influences: blocking of functional groups that are essential to the performance of a biomolecule, displacing other metals found in the system, and changing the conformation of active sites and the quaternary structures of proteins. In at least some ways or under some environmental conditions, many metals are capable of inducing tumors or interacting with genetic materials (Costa *et al.*, 1984; Kazantsis and Lilly, 1986; Norseth, 1988; Woo *et al.*, 1988).

The interaction of a xenobiotic with DNA can damage the chromosomes, cause single- or double-stranded breaks, form DNA adducts, or interfere with the mechanisms involved in repairing these damages. Some of those substances are called aneugenics because they cause changes in the distribution of chromosomes during cell division, leading to numerous chromosomal changes. Some others, called clastogenics, induce breaks and changes in the chromosome structure. For both of these types, it is possible to assess the effects of a certain compound through genotoxicity tests (Rabello-Gay *et al.*, 1991).

The formation of chromosomal abnormalities (CA) is a complex cellular process that is not fully understood, neither at the molecular genetic level nor at the ultrastructural level (Palitti, 1998), despite the fact that CAs are microscopically visible and represent part of a wide range of DNA alterations caused by different mechanisms (Obe *et al.*, 2002). In fish, the hematopoietic tissue found in the kidney is customarily used to obtain mitotic chromosomes (Bertollo *et al.*, 1978) and also to assess chromosomal abnormalities when evaluating genotoxicity (Ale *et al.*, 2004; Cestari *et al.*, 2004; Ferraro *et al.*, 2004; Ramsdorf *et al.*, 2009a).

The comet assay is a fast, sensitive and relatively inexpensive testing tool for the genotoxic potential of chemical substances (Provost *et al.*, 1993; Singh and Stephens, 1997; Belpaeme *et al.*, 1998). In fish, the blood, liver, branchial and renal tissues have been evaluated for genotoxicity using the comet assay (Balpaeme *et al.*, 1998; Ferraro *et al.*, 2004; Ramsdorf *et al.*, 2009, 2012; Ghisi *et al.*, 2011; Benincá *et al.*, 2012; Vicari *et al.*, 2012).

The use of fish as bioindicators allows for the early detection of pollutants in the environment (Frenzilli *et al.*, 2004; Domingos *et al.*, 2009; Katsumiti *et al.*, 2009; Benincá *et al.*, 2012). Fish present several advantages in ecotoxicological studies because they comprise the most diverse group of vertebrates and have a high ecological relevance when exposed to toxic substances. Moreover, fish may present similar results to other vertebrates, humans included (Al-Sabti and Metcalfe, 1995), and are also useful as human food sources (De Camargo and Pouey, 2005; FAO, 2010). The fish species *Rhamdia quelen* (Jundiá) has been used by several researchers as an efficient model for testing the genotoxicity of several classes of xenobiotics (Ghisi *et al.*, 2011; Pamplona *et al.*, 2011). Furthermore, this species is extremely useful and of economic interest in pisciculture in Brazil (Marchioro and Baldisserotto, 1999; Piaia and Baldisseroto, 2000).

The main objective of this study was to evaluate the genotoxic potential of aluminum sulfate in the kidney tissue of *Rhamdia quelen* through subchronic trophic contamination. This method of exposure is considered a realistic model for assessing the effects of xenobiotic contamination of predatory and omnivorous species (Cestari *et al.*, 2004).

## Material and Methods

### Animal maintenance and tissue sampling

*Rhamdia quelen* specimens were obtained from fisheries without any record of contamination and were divided into four groups of 16 fish each (n = 64). The fish were acclimated for approximately 30 days in 250-liter tanks and were later divided into pairs that were kept in 18-liter aquariums for ease of individual feeding. The fish were kept at a temperature of 28°-29° with constant aeration and controlled luminosity (12 h light and 12 h dark). At first, the fish were conditioned for 20 days with single, individual doses of food prepared in blisters using commercial crumb feed and unflavored gelatin (Dr. Oetker). To induce contamination, 5 mg, 50 mg and 500 mg of aluminum sulfate were added to the feed according to the weights of the fish. The fish were fed every three days for 60 days, receiving a total of 20 doses of the does corresponding to each treatment group. The negative control group received only the feed with gelatin in the same way as the groups receiving contaminated feed and the experiments for each group were conducted at the same time.

For tissue sampling, each specimen was anesthetized with 150 mg of benzocaine per L of water (Gontijo *et al.*, 2003), and weighed before and after the bioassays. Furthermore, the fish were measured and sexed. Then, an incision was made from the urogenital pore to the pectoral fin and two 3 mm^3^ piece of the posterior kidney were obtained using tweezers. One was immediately placed in a Petri dish containing 5 mL of culture medium (RPMI solution with 20% of fetal bovine serum and 0.1% of antibiotic-antimycotic) for assessing mitotic metaphases. The second was placed into a microtube filled with 1 mL of fetal bovine serum, which had been kept in ice and in the absence of light, for applying the comet assay technique.

### Tests of chromosomal abnormalities

Metaphases were obtained according to the indirect method described by Fenocchio *et al.* (1991), with some changes as explained here. An approximately 3 mm^3^ piece of the posterior kidney was taken and placed in a Petri dish with 5 mL of culture medium (10.40 g/L RPMI medium 1640, 0.1% of penicillin/streptomycin and the antimycotic Cultilab, and 20% fetal bovine serum, at pH 7.4). The tissue portion was teased apart with tweezers and a glass syringe without a needle to obtain a cell suspension, which was then transferred to tissue culture flasks. The samples were incubated at 29 °C for six hours. Next, 34 μL of colchicine (0.025%) was added to the culture and the samples were incubated for 45 min at 29 °C. The samples were made hypotonic by adding KCl (0.075 M) for 45 min before fixing with a 3:1 methanol/acetic acid solution. Two to three drops of the sample were dropped onto a histological slide that was pre-heated at 54 °C. The slides were air dried and then stained with Giemsa (5% diluted in phosphate buffer, pH 6.8), for 10 min. For each sample, 50 metaphases were analyzed and the chromosomes were visually inspected for possible chromosomal abnormalities.

The comet assay technique was previously described by Speit and Hartmann (1995) and modified by Ramsdorf *et al.* (2009a). The kidney tissue was homogenized at 1500 rpm (homogenizer Potter type) in fetal bovine serum to obtain a cell suspension. Fetal bovine serum is the most appropriate solution for maintaining cell viability for up to 48 h (Ramsdorf *et al.*, 2009b). Moreover, this type of tissue homogenization has been successfully used in several studies (Ramsdorf *et al.*, 2009, 2012; Ghisi *et al.*, 2011; Benincá *et al.*, 2012; Vicari *et al.*, 2012). 40 μL of the cell suspension were mixed with 120 μL of a low melting point (LMP) agarose, and the samples were placed on slides previously covered with a normal agarose layer. The slides were submerged in a lysis solution [Lysis stock solution: NaCl (2.5 M), EDTA (100 mM), Tris (10 mM), NaOH (0.8%), N-lauryl-sarcocinate (1%); Lysis reaction solution: Triton X100 (1%), DMSO (10%), Lysis stock (89%)] for 72 h at 4 °C. Subsequently, the slides were immersed in an alkaline buffer [NaOH (10 N) and EDTA (200 mM), pH 13], for 25 min to induce DNA denaturation. The samples were then submitted to electrophoresis at 300 mA per V/cm for 25 min. The reaction was neutralized using Tris-HCl (0.4 M, pH 7.5, 4 °C) and the samples were fixed in absolute ethanol for 10 min. The slides were stained with 2 μg/mL of ethidium bromide. One hundred nucleoids of each slide were visually categorized using a Leica epifluorescence microscope according to damage rating from 0 (no apparent damage) to 4 (maximum damage) (Collins *et al.*, 1997). The scores were calculated by multiplying the number of nucleoids found in each category and adding the resultant values.

### Statistical analysis

The Kruskal-Wallis non-parametrical test was used with the Student-Newman-Keuls *post-hoc* test. Values of p < 0.05 were considered to be significant.

### Ethical issues

The experiments conducted in this study were approved by the Ethics Committee for Animal Experimentation (CEEA) of the Federal University of Paraná Protocol #23075.053046/2010-33.

## Results

**Figure 1:**
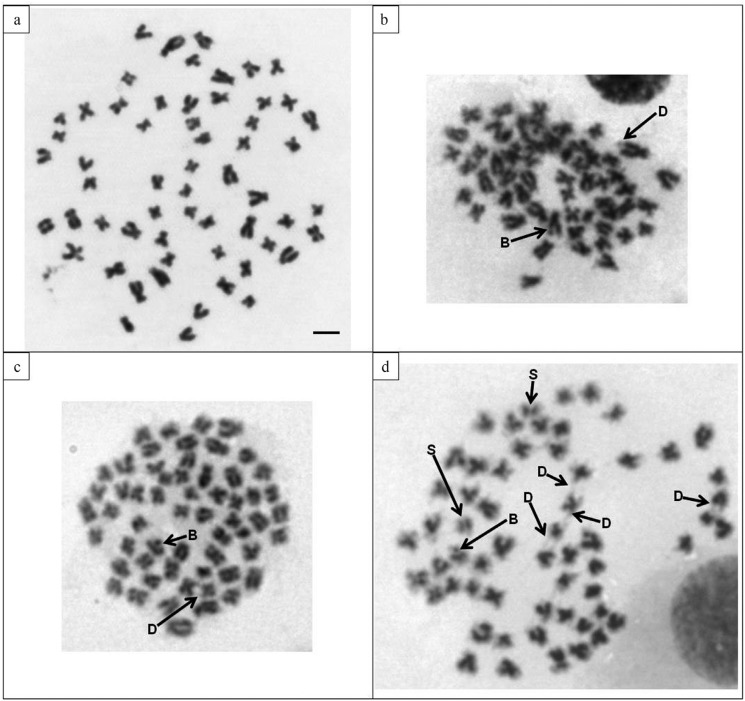
Mitotic chromosomes of *Rhamdia quelen* (2N = 58) submitted to contamination with aluminum sulfate. (a) Metaphase of a specimen from the negative control group without chromosomal abnormalities. (b-d) Metaphases of specimens subjected to 5 mg/kg (b), 50 mg/kg (c) and 500 mg/kg (d). Letter “B” indicates chromatid breaks, “D” indicates decondensation, and “S” the early separation of sister chromatids. Scale bar: 5 μm.

From the 64 specimens of *Rhamdia quelen* used in the bioassay, only 44 (11 specimens from each group) presented mitotic metaphases of sufficient quality for analysis. They showed structural type chromosomal abnormalities (CA) that were categorized as chromatid breaks, DNA decondensation, and early separation of sister chromatids (Figure 1). Abnormal chromosome numbers were not found in any of the groups.

**Figure 2:**
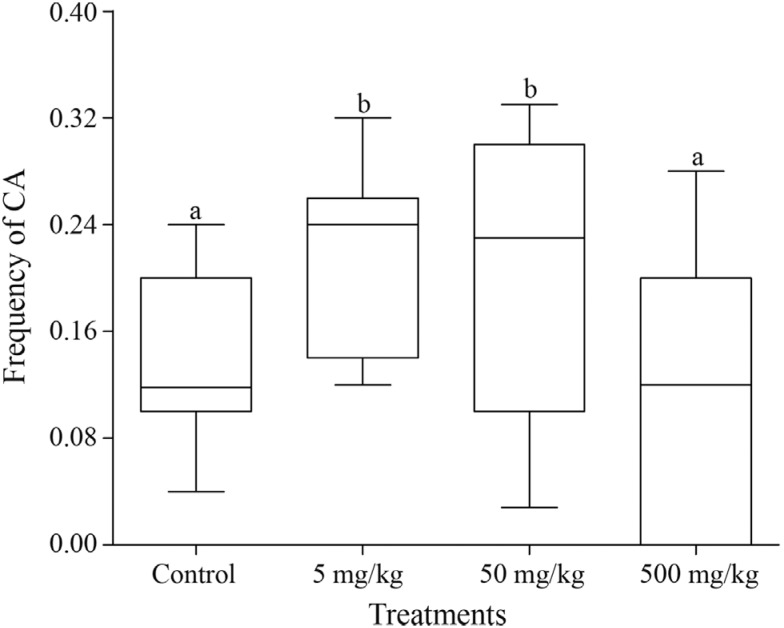
Comparison of the total frequency of chromosomal abnormalities (CA) between the treatments. The box and whisker plots show the median and the first and third quartiles. Different letters indicate statistically significant differences (p < 0.05).

A total of 1,966 metaphases were analyzed, which included 561 in the negative control group, 531 in the 5 mg/kg group, 470 in the 50 mg/kg group and 404 in the 500 mg/kg group. The total frequency of CAs was significantly higher in the groups contaminated with 5 mg/kg (p = 0.0154) and 50 mg/kg (p = 0.0245) of aluminum sulfate compared to the negative control group. The frequencies of CAs were similar in the 500 mg/kg group and the negative control (p > 0.05) (Figure 2).

**Figure 3:**
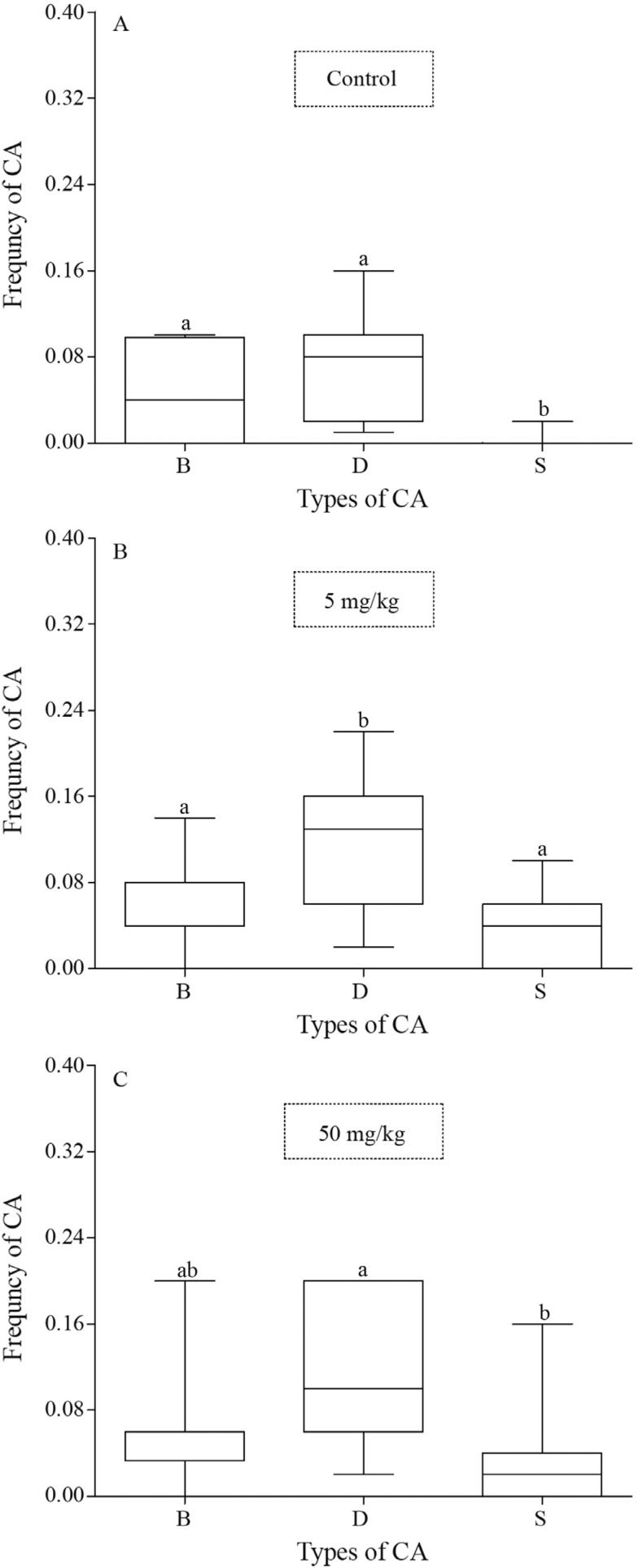
Comparison of the frequency of the types of chromosomal abnormalities (CA). (A) Negative control group; (B) group contaminated with 5 mg/kg of aluminum sulfate; and (C) group contaminated with 50 mg/kg. The box and whisker plots show the median and the first and third quartiles. Different letters indicate statistically significant differences (p < 0.05). Types of CA: B = breaks; D = decondensation; S = early separation of the sister chromatids.

With regard to the types of CAs found in each treatment, the negative control group showed a higher frequency of chromatid breaks and decondensation relative to the early separation of sister chromatids (p = 0.0055, p < 0.0001, respectively) (Figure 3A). In the group contaminated with 5 mg/kg of aluminum sulfate, the frequency of decondensation was significantly higher than the frequency of either chromatid breaks or separations (p = 0.0206, p = 0.0023, respectively) (Figure 3B). The group contaminated with 50 mg/kg showed differences between the frequencies of decondensation and chromatid separation (p = 0.0014) (Figure 3C). There was no difference among the types of CA in the group contaminated with 500 mg/kg (p > 0.05).

**Figure 4:**
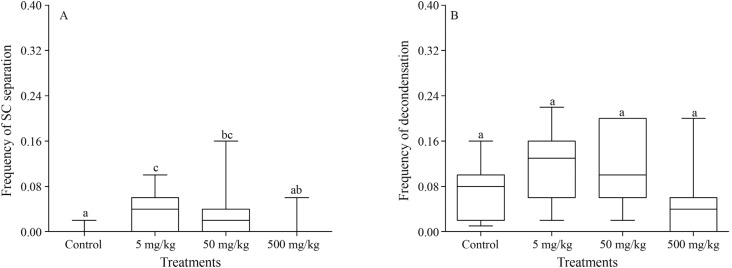
Effect of the aluminium sulfate concentrations on sister chromatid separation and chromatin decondensation. (A) Comparison of the frequency of the early separation of the sister chromatids across the treatments. (B) Comparison of the frequency of the decondensation across the treatments. The box and whisker plots show the median and the first and third quartiles. Different letters indicate statistically significant differences (p < 0.05).

The analysis of each type of CA showed that, relative to the control group, only the early separation of sister chromatids was significantly higher in the groups treated with 5 mg/kg and 50 mg/kg aluminum sulfate (p = 0.0099, p = 0.0329, respectively) (Figure 4A). However, the groups contaminated with 5 mg/kg and 50 mg/kg tended to present differences in the decondensation frequency (p = 0.0570) (Figure 4B).

**Figure 5:**
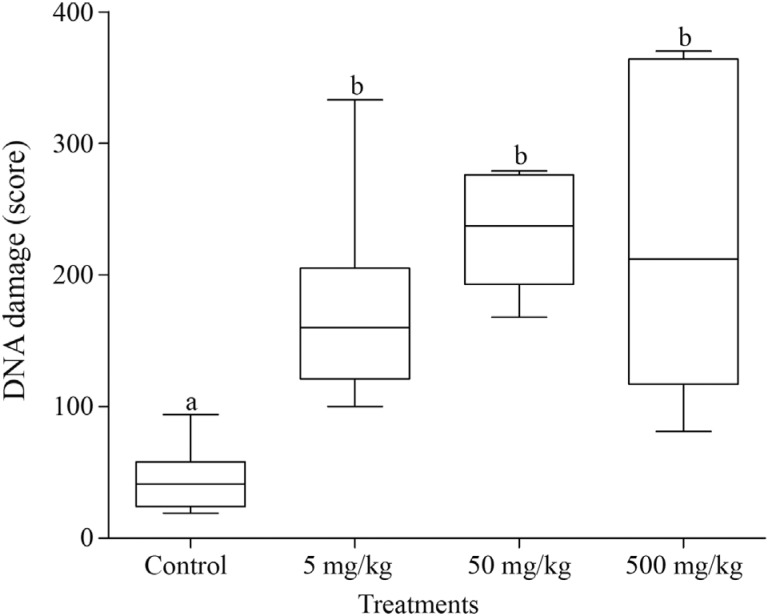
Comparison of the DNA damage scores (comet assay) in the kidney tissue cells between the treatments. The box and whisker plots show the median and the first and third quartiles. Different letters indicate statistically significant differences (p < 0.05).

The comet assay showed significantly higher damage scores in the groups treated with 5 mg/kg, 50 mg/kg and 500 mg/kg compared to the negative control group (p = 0.001, p < 0.0001, p < 0.0001, respectively). This finding confirms how sensible and easy it is to obtain the results through this genotoxic technique when compared to the CA technique. Nonetheless, no dose-response relationship was observed because the comet assay showed no increase in the amount of DNA damage with increasing doses (Figure 5).

## Discussion

There is prior evidence that aluminum leads to chromosomal abnormalities, micronuclei and the exchange of sister chromatids in human lymphocytes (Roy *et al.*, 1990; Migliore *et al.*, 1999; Banasik *et al.*, 2005), and contamination by aluminum and fluoride has been shown to cause telomeric damage in cultured human lymphocytes. Damage in the telomeric region caused by toxic compounds leads to chromosomal instability, resulting in cell death as a consequence (Patel *et al.*, 2009).

In fish, different CA types have been described following exposure to metal contaminants, such as lead and tributyltin (Cestari *et al.*, 2004; Ferraro *et al.*, 2004., Ale *et al.*, 2004; Ramsdorf *et al.*, 2009a), leading us to suggest that the decondensation of the telomeric regions of the chromosomes may have occurred due to structural modifications to the proteins involved in DNA compaction, thus making the DNA more susceptible to breaks. This may have also caused the chromatid breaks. Telomeric decondensation and chromatid breaks have been described for contaminations with lead in trophic bioassays conducted for 60 days in the fish species *Hoplias malabaricus* (Erythrinidae) (Ferraro *et al.*, 2004; Cestari *et al.*, 2004). The centromere is a region of the chromosome with compact heterochromatin, in which the histones are responsible for the compaction process (Hagele, 1977; Michailova *et al.*, 1997). In the species *Chironomus riparius* (Diptera, Chironomidae), decondensation of the centromeric region observed in response to to aluminum contamination was attributed to the inhibition of histone synthesis (Michailova *et al.*, 2003).

The early separation of sister chromatids observed in this work may have occurred due to changes to the proteins that keep the sister chromatids in cohesion, or that facilitate the mobility of the chromosomes. Using yeast, Michaelis *et al.* (1997) showed that cohesins are responsible for the cohesion of sister chromatids during the cell cycle. In cells containing mutant forms of the proteins SCC1, SCC2, SMC1 and SMC3, the sister chromatids undergo an early separation. The SCC1 protein connects to the chromosome in the final stage of the G1 phase and remains connected to the sister chromatids until metaphase. Moreover, other reports have shown that aluminum affects chromosomal mobility during the cell division process (Lukiw and Mclachlan, 1995).

Several studies discussed by Durante *et al.* (2013) have shown that chromosomal abnormalities, such as chromatid breaks, chromosomal breaks or rearrangements, are caused by failures in the repair system when there is a double-stranded break in the DNA. Nonetheless, the exact mechanisms are not yet clear.

The comet assay showed the genotoxicity of aluminum, even though the damage detected may have been subjected to DNA repair mechanisms. Aluminum toxicity has been studied in the mononuclear leukocytes of people who used aluminum utensils on a daily basis to cook or store food. In that study, high levels of DNA damage were related to the generation of oxidative stress (Celik *et al.*, 2012). In another study, the comet assay was used to check the genotoxic potential of aluminum in the fish *Prochilodus lineatus* contaminated by acute, subchronic exposure in water. An increase in the DNA damage in erythrocytes was observed after 6 h and 96 h of exposure, whereas there were no differences relative to the negative control after 24 h and 15 days. These results indicated that the DNA underwent a damage repair process (Galindo *et al.*, 2010).

In the present work, the comet assay was used to confirm the genotoxic effects observed in the CA test. Ramsdorf *et al.* (2009a) used the comet assay to verify the DNA damage seen in *Hoplias malabaricus* subjected to inorganic lead contamination by intraperitoneal injection. Benincá *et al.* (2012) also demonstrated DNA damage in this tissue by monitoring the Santa Marta and Camacho Lakes (Santa Catarina Coast-Southern of Brazil). It is known that the comet assay can detect DNA damage, including both single- and double-stranded breaks, in individual cells (Singh *et al.*, 1988; Fairbairn *et al.*, 1995; Sazaki *et al.*, 1999). This property makes it a very useful assay for several tissues. In line with observations from other genotoxicity studies (Ghisi *et al.*, 2011; Pamplona *et al.*, 2011), we could confirm this in the present study, showing that *Rhamdia quelen* appears to be a good test organism for genotoxicity assays.

In conclusion, we demonstrated that aluminum sulfate was genotoxic in the kidney tissue cells of *Rhamdia quelen*. The test for chromosomal abnormalities showed clastogenic effects, whereas the comet assay confirmed the genotoxic potential of aluminum sulfate. Even at very low doses, the comet assay was highly effective at showing DNA damage, and the CA test was shown to be an efficient biomarker in sub-chronic bioassays. In our future works, we will evaluate the damage to other tissues of *Rhamdia quelen* and *Hoplias intermedius* subjected to trophic aluminum sulfate contamination. Also, additional studies must be conducted to investigate the mechanisms that cause the chromosomal abnormalities.
